# Anti-CD19 chimeric antigen receptor T-cell therapy for adult Philadelphia chromosome-positive acute lymphoblastic leukemia: Two case reports

**DOI:** 10.1097/MD.0000000000005676

**Published:** 2016-12-23

**Authors:** Yang-min Zhu, Zhao Wu, You-ping Tan, Yuan-yuan Du, Zhi Liu, Rui-ming Ou, Shuang Liu, Cheng-fei Pu, Jing Jiang, Jin-ping Wang, Lei Xiao, Qing Zhang

**Affiliations:** a Department of Hematology, Guangdong No. 2 Provincial People's Hospital, Guangzhou; b Innovative Cellular Therapeutics Co., Ltd. (Formerly SiDanSai Biotechnology Co., Ltd), Shanghai, China.

**Keywords:** acute lymphoblastic leukemia, CD19, chimeric antigen receptor, cytokine release syndrome, Philadelphia chromosome

## Abstract

**Rationale:**

The presence of the Philadelphia chromosome (Ph) in acute lymphoblastic leukemia (ALL) has been associated with a high risk of disease relapse and a poor prognosis. Allogeneic hematopoietic stem cell transplantation (HSCT) is an established treatment for adults with Ph-positive ALL, but relapse remains the primary cause of treatment failure, and is associated with an extremely poor prognosis. The emergence of resistance to tyrosine kinase inhibitors (TKIs) poses a challenge for patients with disease relapses after initial treatment with TKI-containing regimens.

**Patient concerns:**

Two patients with TKI-resistant recurrent Ph-positive ALL.

**Diagnoses:**

Ph-positive ALL.

**Interventions:**

Anti-CD19 CAR T-cell infusion.

**Outcomes:**

One patient's bone marrow blasts decreased significantly, and the other reached negative minimal residual disease (MRD). However, we first recorded the development of new-onset acute graft-versus-host disease (aGVHD) after anti-CD19 CAR T-cell infusion in a patient who received allogeneic HSCT. Our 2 case reports also demonstrate the efficacy of anti-CD19 CAR T-cell therapy in the treatment of TKI-resistant Ph-positive ALL.

**Lessons:**

Our report suggests that anti-CD19 CAR T-cell therapy may be a promising option for the treatment of relapsed Ph-positive ALL after conventional chemotherapy or allogeneic HSCT. However, caution is due given the possibility of the adverse effects of cytokine release syndrome (CRS)-induced aGVHD for patients receiving allogeneic HSCT.

## Abstract 2

Supplemental Digital Content is available in the text

## 1 Introduction

The Philadelphia chromosome (Ph) is the most common cytogenetic abnormality associated with adult acute lymphoblastic leukemia (ALL), occurring in 20% to 40% of patients.^[1]^ The presence of the t(9; 22) chromosomal translocation is the single most important adverse prognostic factor in Ph-positive ALL, with long-term survival of less than 20% with current chemotherapy regimens.^[1]^ Tyrosine kinase inhibitors (TKIs) directed against the ABL kinase are now used routinely during first-line therapy for Ph-positive ALL and result in hematologic remission rates exceeding 90%.^[2,3]^ Although outcomes have improved substantially with TKI-based regimens versus with historic controls, allogeneic hematopoietic stem cell transplantation (HSCT) at the first remission provides the best chance of a cure for the majority of patients eligible for HSCT.^[3]^ However, disease recurrence remains the main cause of failure. Treatment options are extremely limited for patients with Ph-positive ALL who experience relapse after receiving allogeneic HSCT. In nontransplanted patients, the emergence of resistance to TKI therapy also poses a challenge for patients with disease relapse after initial treatment with TKI-containing regimens.

Chimeric antigen receptors (CARs) are fusion proteins that incorporate an antigen- recognition moiety and a T-cell activation domain.^[4]^ T-cells can be modified genetically to express anti-CD19 CARs on their surface and have been shown to exert cytotoxic effects against CD19-positive B-cells. Both autologous and allogeneic anti-CD19 CAR T-cells have produced remission in previously treated patients with B-cell malignancies.^[5,6]^ We describe 2 patients with Ph-positive ALL resistant to TKIs who underwent anti-CD19 CAR T-cell infusions. One patient's bone marrow blasts decreased significantly, and the other reached negative minimal residual disease (MRD) status.

## 2 Case reports

**Figure 1:**
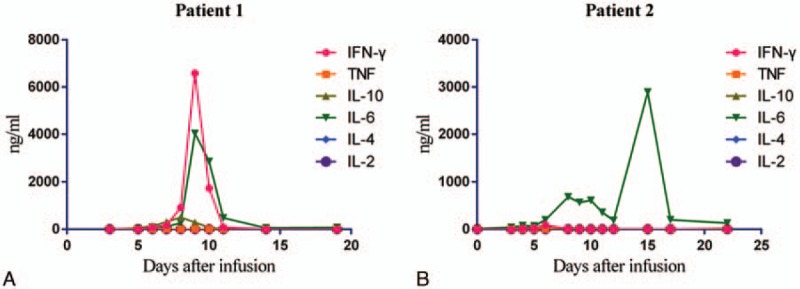
Serum interleukin-6 (IL-6) levels increased after anti-CD19 CAR T-cell infusion.

**Figure 2:**
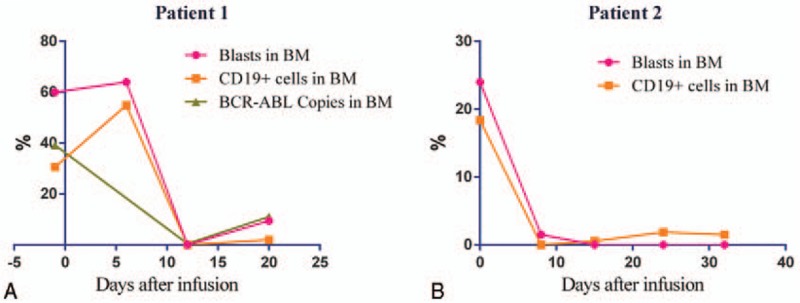
Anti-CD19 chimeric antigen receptor (CAR) T-cells are effective against tyrosine kinase inhibitor (TKI)-resistant Philadelphia chromosome (Ph)-positive acute lymphoblastic leukemia (ALL).

Patient 1 was a 39-year-old woman who presented to a local hospital for systemic subcutaneous ecchymosis and nasal bleeding on January 13, 2015. Blood examination revealed a white blood cell (WBC) count of 31.96 × 10^9^/L, hemoglobin (HGB) of 85 g/L, and a platelet (PLT) count of 10 × 10^9^/L. Bone marrow examination and flow cytometry suggested B-cell ALL. Cytogenetics revealed the Philadelphia chromosome and the *BCR-ABL* fusion gene was positive. She was thus diagnosed with Ph-positive ALL. The patient was given induction chemotherapy with the vincristine, daunorubicin, L-asparaginase, prednisone, and cyclophosphamide (VDCLP) protocol in combination with oral administration of imatinib mesylate capsules (0.4 g/day) on January 21, 2015. She was then discharged after hematopoietic recovery. However, the patient stopped taking imatinib mesylate capsules on her own accord in April 2015. On June 20, 2015, she was admitted to our hospital. At presentation, her physical examination showed multiple enlarged superficial lymph nodes in the neck, armpits, and groin (the largest was ∼2 × 3 cm). Blood examination revealed a WBC count of 194.49 × 10^9^/L, HGB of 78 g/L, and PLT of 18 × 10^9^/L. Bone marrow examination revealed 91% lymphoblasts. Bone marrow fluorescent in situ hybridization (FISH) detected a positive *BCR-ABL* fusion gene (positive rate = 97%). Bone marrow quantitative real-time polymerase chain reaction (QRT-PCR) detected a positive BCR-ABL p190 transcript (BCR-ABL/ABL, 47.7%). Bone marrow Sanger sequencing found T315I and E355G mutations in the ABL kinase region of the *BCR-ABL* fusion gene. The patient was given prednisone after admission; WBCs declined gradually, and the enlarged lymph nodes regressed significantly. Subsequently, 150 mL of peripheral blood was used to prepare anti-CD19 CAR T-cells. Lymphodepleting chemotherapy with the FC regimen (cyclophosphamide 60 mg/kg, days −8 to −7; fludarabine 25 mg/m^2^, days −6 to −4) was given on July 5, 2015. On day −1, 3 days after chemotherapy, the patient exhibited persistent disease with 60% blasts present in the bone marrow. Then, she received an infusion of anti-CD19 CAR T-cells that had been expanded with anti-CD3 and anti-CD28 antibodies and lentivirally transduced to express the anti-CD19 CARs (Innovative Cellular Therapeutics Co., Shanghai, China). The total dose was 1.19 × 10^6^ CAR-positive T-cells/kg (transduction efficiency was 40%), given over a period of 3 consecutive days. No immediate infusion-related toxic effect was noted, but she developed a febrile syndrome, with rigor and transient hypotension by days +5 to +8, cytokine levels (Fig. 1A), C-reactive protein (CRP 161.3 mg/L), and ferritin (139,355.4 ng/mL) increased significantly, anti-infection treatment was ineffective, indicating Grade 2 cytokine release syndrome (CRS) according to the University of Pennsylvania grading system. Tocilizumab (8 mg/kg) was given on day +8 after infusion; within hours, the patient's body temperature dropped to normal. On July 28, 2015 (day +12), the patient's bone marrow blasts had decreased significantly (Fig. 2A). Flow cytometry of bone marrow cells detected MRD of 0.06%. Positive BCR-ABL p190 transcript (0.5%) was detected by QRT-PCR. On August 9, 2015 (day +24), the patient presented with central nervous system (CNS) symptoms of shallow left frontal pain and left hypoplasia. Lumbar puncture revealed a cerebrospinal fluid (CSF) pressure of 250 mmH_2_O. We did not detect anti-CD19 CAR T-cells in the CSF because of the many prolymphocytes in the CSF smear. She was diagnosed with CNS leukemia (CNSL) (Figure 5; Data Supplement). The patient received CNS-directed intrathecal chemotherapy followed by multicourse systemic chemotherapy. She achieved a second morphologically complete remission, and then accepted allogeneic HSCT from a sibling donor. She is still alive and in follow-up.

**Figure 3:**
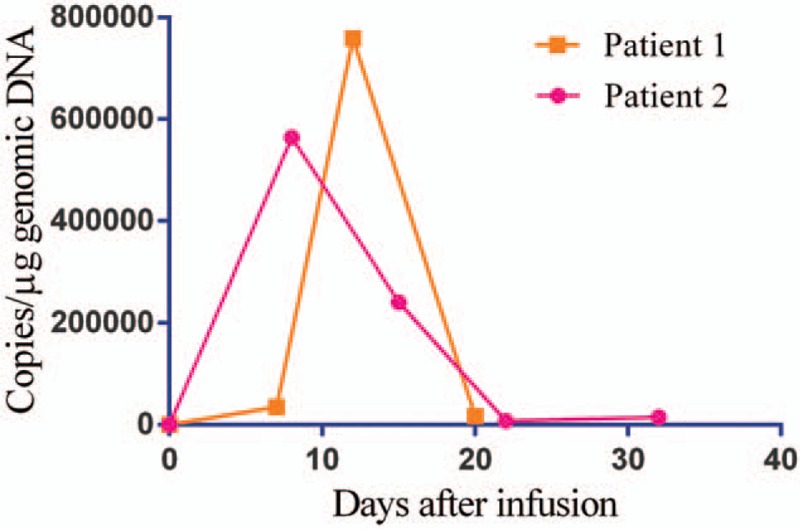
Expansion of anti-CD19 CAR T-cells in peripheral blood.

**Figure 4:**
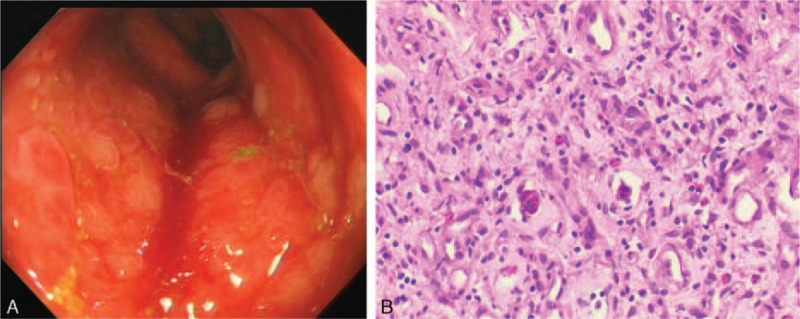
Electronic colonoscopy revealed mucosal congestion and edema throughout the large intestine. The pathological examination suggested chronic mucosal inflammation.

Patient 2 was a 29-year-old man who presented at a local hospital with fever and ostealgia on December 30, 2012. Blood examination revealed a WBC count of 18.04 × 10^9^/L, HGB of 135 g/L, and PLT of 98 × 10^9^/L. A bone marrow examination revealed the presence of t(9; 22) (q34; q11) in a cytogenetic evaluation, and a positive BRC-ABL p190 transcript was detected by QRT-PCR. He was thus diagnosed with Ph-positive ALL. The patient was given chemotherapy with the VDLP protocol in January 2013, and the symptoms were alleviated. He then received multiple consolidated chemotherapies with vincristine, daunorubicin, cyclophosphamide, and prednisone (VDCP), hyper-CVAD-A, hyper-CVAD-B, VDP, and hyper-CVAD-B regimens, followed by haploidentical allogeneic HSCT on September 12, 2013. During the HSCT, he received infusions of bone marrow and peripheral blood stem cells (mononuclear cells 7.08 × 10^8^/kg, CD34^+^ cells 3.02 × 10^6^/kg). In addition, 31 mL of umbilical cord blood (total nucleated cells 1 × 10^7^/kg) was infused on September 25, 2013. During the HSCT, the patient had a history of intestinal acute graft-versus-host disease (aGVHD), but this resolved with anti-rejection treatments. However, flow cytometry of bone marrow cells detected positive MRD 6 months after transplantation. The patient was given targeted therapy with imatinib mesylate, but 2 weeks later, the MRD was found to have progressed. He then received chemotherapy with the VDCP protocol, but he was still MRD-positive. Due to imatinib resistance, therapy with dasatinib was started and MRD-negative status was achieved. However, the patient stopped dasatinib therapy for 1 month due to the adverse reaction of pleural effusion. Positive BCR-ABL p190 transcript was detected (0.29%) in bone marrow cells by QRT-PCR on April 7, 2015. Positive BCR-ABL p190 transcript was still detected (1.95%) by QRT-PCR on June 10, 2015. The patient chose to continue dasatinib therapy. Positive MRD was still detected by flow cytometry (0.78%) and QRT-PCR (4.74%) in bone marrow cells on June 23, 2015. Chemotherapy was given to prevent recurrence on June 25, 2015, but the patient did not finish the chemotherapy due to infection. Then, cryopreserved donor peripheral blood stem cells (1 × 10^7^/kg) were infused on June 30, 2015. The patient was admitted to our hospital on August 17, 2015. Blood examination revealed a WBC count of 3.6 × 10^9^/L, HGB of 88 g/L, and PLT of 77 × 10^9^/L. Flow cytometry of bone marrow cells revealed 26% abnormal immature cells, with a B-cell phenotype accompanied by myeloperoxidase (MPO) expression. STR examination of bone marrow cells revealed incomplete donor chimerism (87% of donor origin). Peripheral blood mononuclear cells (PBMCs, 100 mL) were collected to prepare anti-CD19 CAR T-cells on August 25, 2015. Lymphodepleting chemotherapy with the FC regimen (cyclophosphamide 60 mg/kg, day −8; fludarabine 25 mg/m^2^, days −4 to −3) was given on August 29, 2015. However, he developed a high fever and hypotension during the chemotherapy; we then reduced the chemotherapy dose. After anti-infection and rehydration treatment, the patient's body temperature returned to normal, and the hypotension resolved. On day −1, 2 days after chemotherapy, the patient exhibited persistent disease with 24% blasts present in bone marrow. Then, anti-CD19 CAR T-cells were infused at a total dose of 1.0 × 10^6^ CAR-positive T-cells/kg (transduction efficiency was 30%) in a single dose. No immediate infusion-related toxic effect was noted, but he developed a fever and hypotension on day +1. Anti-infection treatment was ineffective, with increased cytokine levels (Fig. 1B), CRP (107.5 mg/L), and ferritin (15,570.1 ng/mL), indicating grade 4 CRS. Tocilizumab (8 mg/kg) was given on day +6 after the infusion; the patient's body temperature dropped to normal quickly. On day +8, a bone marrow myelogram revealed immature lymphocytes accounting for 1.5%, and abnormal lymphocytes accounting for 14.5% (considering CAR T-cells). Both flow cytometry and QRT-PCR of bone marrow cells indicated MRD-negative status (Fig. 2B). During this time period, anti-CD19 CAR T-cells revealed rapid expansion in the peripheral blood (Fig. 3). However, the patient developed abdominal pain and diarrhea on September 15, 2015 (day +9), and the symptoms became aggravated gradually, with diarrhea reaching more than 20 times per day. Cytomegalovirus DNA in blood and multiple stool bacterial cultures were negative. Anti-diarrheal and anti-infection treatments were ineffective. STR of bone marrow cells revealed full donor chimerism on September 28, 2015. An electronic colonoscopy revealed mucosal congestion and edema of the whole large intestine (Fig. 4A). A pathological examination suggested mucosal chronic inflammation, surface erosion, and granulation tissue hyperplasia. There was more inflammatory cell infiltration in the mesenchyme (Fig. 4B). A diagnosis of intestinal aGVHD was made in accordance with these clinical and histopathological manifestations. The patient was given anti-rejection treatment, with methylprednisolone, methotrexate, and cyclosporine for 3 weeks. His symptoms improved gradually; he was kept on maintenance treatment with cyclosporine for 1 month. On October 8, 2015 (day +32), the patient was MRD-negative in terms of bone marrow cells by flow cytometry and QRT-PCR (Figure 6; Data Supplement). At the last follow-up (6 months after infusion), he was in complete molecular remission and still alive.

## 3 Discussion

We report here 2 cases of adult patients with recurrent Ph-positive ALL who underwent anti-CD19 CAR T-cell therapy. Sanger sequencing showed the T315I mutation in the ABL kinase region of the *BCR-ABL* fusion gene in 1 case. The patient was resistant to first- and second-generation TKIs, and routine chemotherapy was unsatisfactory. After infusion of CAR T-cells, the patient's bone marrow blasts decreased significantly, but this was not complete remission, probably due to the modest doses of cells administered in our treatment plan. This is the first time we demonstrated the efficacy of anti-CD19 CAR T-cell therapy in the treatment of TKI-resistant Ph-positive ALL. Although the patient did not receive a second infusion, due to the CNSL, complete molecular remission or MRD-negative status can reasonably be expected with a second or multiple infusions of anti-CD19 CAR T-cells.

The other case showed recurrence after allogeneic HSCT. The disease was still aggravated during treatment with a second-generation TKI, which could also be considered TKI resistance. CRS developed after infusion of anti-CD19 CAR T-cells, which induced aGVHD. The symptoms were alleviated after tocilizumab and anti-rejection treatments. This is the first reported CRS-induced aGVHD after the treatment with CAR T-cells in an adult Ph-positive ALL patient with recurrence after allogeneic HSCT.

It has been reported that allogeneic anti-CD19 CAR T-cells can effectively treat B-cell malignancies that progress after allogeneic HSCT without developing new-onset aGVHD.^[7]^ In patient 2, STR of bone marrow cells revealed that most of the cells originated from the donor, and aGVHD is unlikely to occur after infusion of chimeric anti-CD19 CAR T-cells. One factor that may explain this lack of aGVHD is the limited persistence of anti-CD19 CAR T-cells, because aGVHD takes a median of 4 weeks to develop after standard donor lymphocyte infusion (DLI), whereas the highest levels of anti-CD19 CAR T-cells were noted before 4 weeks postinfusion and declined thereafter.^[6]^ Another possible explanation is the modest dose administered to our patients, which was not sufficient to induce aGVHD, versus standard DLI. However, aGVHD was indeed observed in combination with CRS in 1 of our patients. The most likely explanation seems to be CRS-induced aGVHD. The pathophysiology of aGVHD has been considered to involve a 3-step process: first, host tissue damage due to the conditioning regimen, infections, and possibly the underlying disease, which induces the secretion of inflammatory cytokines; second, donor T-cell activation and proliferation under the stimulation of increased cell surface expression of leukocyte adhesion molecules and human leukocyte antigen (HLA) molecules, followed by a strong cytokine response (such as the secretion of IL-2 and IFN-γ); and third, these cytokines further promote antigen presentation and the recruitment of effector T-cells and innate immune cells, which further augment the inflammatory cytokine response (such as TNF).^[8]^ The effector T-cells, natural killer (NK) cells, macrophages, and inflammatory cytokines result in end-organ damage. Acute GVHD can occur in the absence of primary tissue injury in settings such as transfusion-related GVHD, especially in the case of a greater HLA disparity between donor and host. This indicates that the conditioning phase is not absolutely necessary for the induction of aGVHD. However, the network of soluble cytokines form a key link between each of the 3 steps and is responsible for the bulk of target organ damage.^[9]^ Thus, we suspect that aGVHD was induced by CRS on the basis of direct tissue damage due to lymphodepleting chemotherapy and a greater HLA disparity between the donor and host.

There was still no protocol for subsequent treatment after anti-CD19 CAR T-cell therapy for B-ALL; 2 or more infusions, allogeneic HSCT, or follow-up could be considered. Decisions should be made according to a comprehensive judgment with persistence of CAR T-cells, MRD, and STR. For patients with recurrence after allogeneic HSCT, induction of graft-versus-leukemia (GVL) immune surveillance could be considered to prevent recurrence.^[10]^ For patients with Ph-positive ALL, maintenance treatment with TKIs, if not resistant, could be considered after negative MRD status is reached.^[11]^

## 4 Conclusion

Our case reports demonstrate the efficacy of anti-CD19 CAR T-cell therapy in the treatment of TKI-resistant adult Ph-positive ALL. They also suggest that anti-CD19 CAR T-cell therapy may be a promising option for the treatment of relapsed Ph-positive ALL after conventional chemotherapy or allogeneic HSCT. However, caution should be paid to the possibility of the adverse effect of CRS-induced aGVHD for patients receiving allogeneic HSCT. The optimal dose of CAR T-cells and the follow-up treatment remain to be clarified. This is a report of 2 cases; further well-designed and randomized studies with larger numbers of cases are needed to fully evaluate this strategy.

## 5 Methods

### 5.1 Anti-CD19 CAR T-cell manufacturing

We designed a CAR lentivirus vector that consisted of an FMC63-derived CD19-specific single-chain variable fragment (scFv), a 4–1 BB costimulatory domain, and a CD3ζ signaling domain.^[12–14]^ The lentivirus was produced by transfecting 293T cells with CAR lentiviral vectors and viral packaging plasmids. Peripheral blood was collected from the patient, and CD3 T cells were isolated and activated as described in.^[15]^ CD3 T cells were cultured in X-VIVO 15 medium (Lonza Group Ltd., Switzerland) containing 100 U/mL interleukin-2 (IL-2), and transduced with CAR lentivirus supernatant within 24 to 48 hours. Transduced anti-CD19 CAR T-cells were maintained at about 0.5 to 1 × 10^6^/mL before infusion. Transduction efficiency was evaluated at 5 to 7 days after CAR lentivirus transduction, and quality controls for fungi, bacteria, mycoplasma, chlamydia, and endotoxin were performed by a third-party medical laboratory (KingMed Diagnostics, China). On the day of infusion, fresh anti-CD19 CAR T-cells were delivered to the hospital by hand in the shortest time. The CD3/CD28 beads were removed and the cells for fresh infusion were washed and concentrated in 20 mL infusion media that contained Plasma-Lyte A (Baxter Healthcare Corporation, USA) along with 5% human serum albumin (CSL Behring, USA). A quality control assessment of the infusion cells was also performed. Flow cytometry was used to characterize surface expression of anti-CD19 CAR T-cells with antibodies of the anti-hCD3 FITC, anti-hCD19 APC, anti-hCD45 PE, and PE streptavidin (BD Bioscience, USA). The transduction efficiency was determined by FACS using goat-anti-mouse F(ab’)2 antibody (Jackson Immuno Research, USA).^[16]^

### 5.2 Assessment of anti-CD19 CAR T-cells persistence

Serial peripheral blood samples after the anti-CD19 CAR T-cells infusion were collected in K_2_EDTA BD Vacutainer tubes (BD Bioscience). Persistence of anti-CD19 CAR T-cells in patients was determined by FACS using goat-anti-mouse F(ab’)2 antibody (Jackson Immuno Research). Circulating CAR T-cell numbers per μL were calculated on the basis of measured absolute CD3^+^ T lymphocyte counts. Genomic DNA was extracted using a QIAamp DNA Blood Mini Kit (Qiagen, Germany). Quantitative real-time PCR was performed in triplicate using the ABI 2 × Taqman Universal Master Mix with the AmpErase UNG (Applied Biosystems, USA), in a 7500 real-time PCR system (Applied Biosystems). Copy numbers per microgram of genomic DNA were calculated from a standard curve of 10-fold serial dilutions of purified CAR plasmid containing 10^2^ to 10^8^ copies/μL. Amplification of an internal control gene was used for normalization of DNA quantities. Primers/probes specific for the anti-CD19 CAR transgene and an internal control gene were as described before.^[15]^

### 5.3 Cytokine detection

The levels of IL-2, IL-4, IL-6, IL-10, tumor necrosis factor (TNF), and interferon-gamma (IFN-γ) in the peripheral blood were detected using flow cytometry and the Cytometric Beads Array Flex Set (BD Bioscience).

## Acknowledgment

We thank Guiming Wang from the Department of Pathology for providing pathological pictures.

## Supplementary Material

Supplemental Digital Content
