# Supplementary figures and images for: Anti-CD19 chimeric antigen receptor T-cell therapy for adult Philadelphia chromosome-positive acute lymphoblastic leukemia: Two case reports
Source: Medicine (Baltimore). 2016 Dec 23;95(51):e5676. doi: 10.1097/MD.0000000000005676 (PMC5181821; doi:10.1097/MD.0000000000005676)

Figure 5. Timeline chart for patient 1.


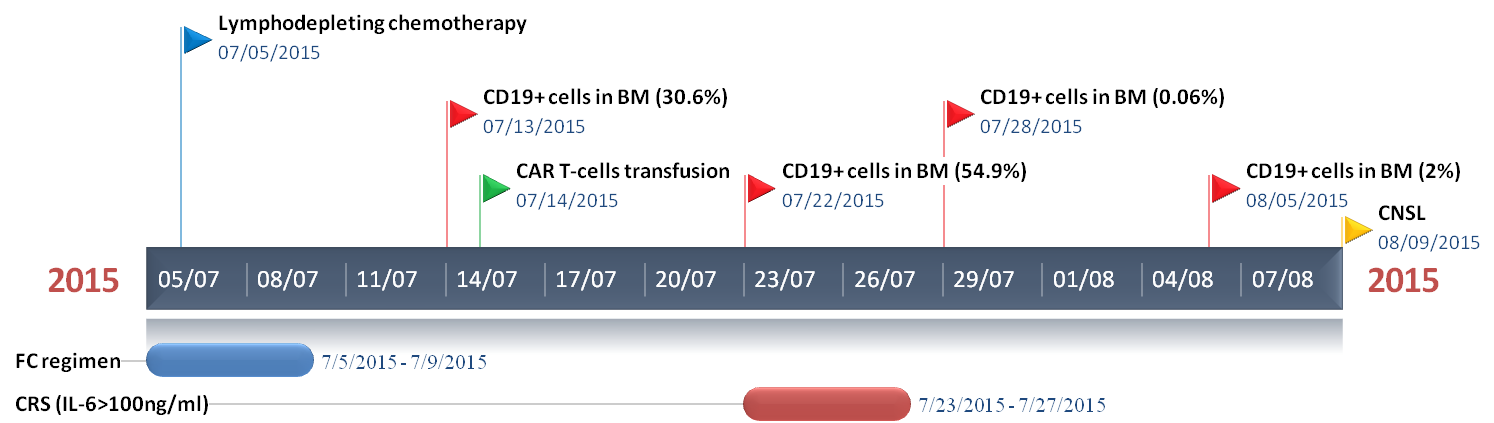


Figure 6. Time line chart for patient2.


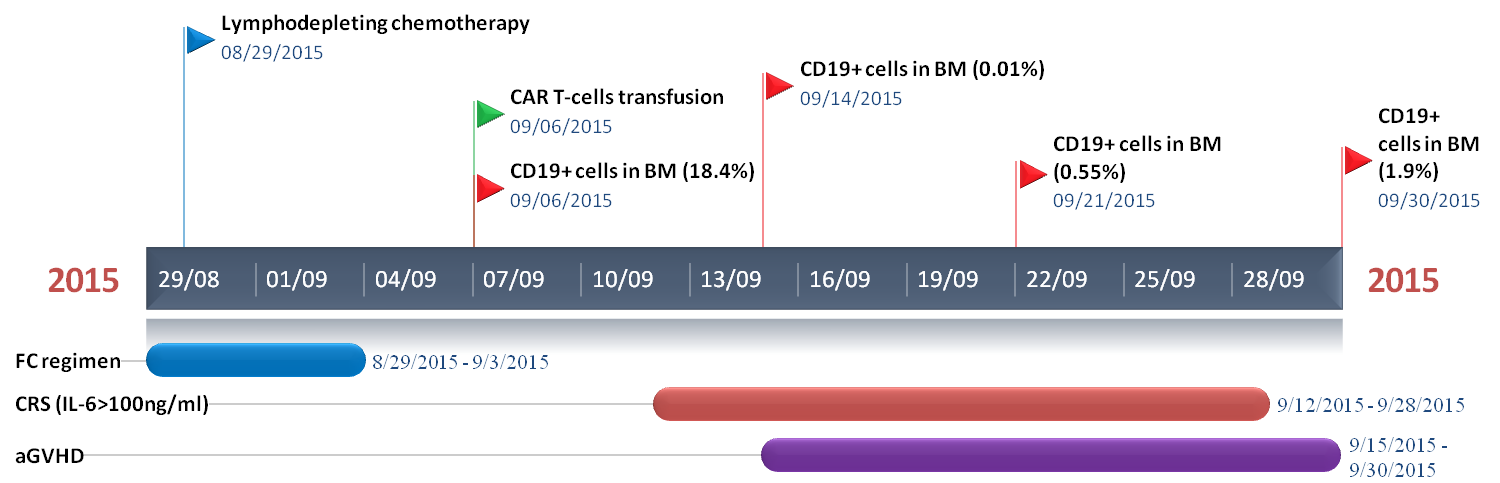

Supplement: Supplemental Digital Content [file medi-95-e5676-s001.doc]
